# Ligand Activation of TAM Family Receptors-Implications for Tumor Biology and Therapeutic Response

**DOI:** 10.3390/cancers8120107

**Published:** 2016-11-29

**Authors:** Viralkumar Davra, Stanley G. Kimani, David Calianese, Raymond B. Birge

**Affiliations:** Department of Microbiology, Biochemistry and Molecular Genetics, Rutgers New Jersey Medical School, Newark, NJ 07103, USA; davravr@gsbs.rutgers.edu (V.D.); kimanisg@njms.rutgers.edu (S.G.K.); dcc139@gsbs.rutgers.edu (D.C.)

**Keywords:** Tyro3, Axl, Mertk, Gas6, protein S, immune evasion, tumor microenvironment

## Abstract

The TAM family of receptors (i.e., Tyro3, Axl, and Mertk), and their ligands Growth arrest specific factor 6 (Gas6) and Protein S (Pros1) contribute to several oncogenic processes, such as cell survival, invasion, migration, chemo-resistance, and metastasis, whereby expression often correlates with poor clinical outcomes. In recent years, there has been great interest in the study of TAM receptors in cancer, stemming both from their roles as oncogenic signaling receptors, as well as their roles in tumor immunology. As a result, several classes of TAM inhibitors that include small molecule tyrosine kinase inhibitors, monoclonal antibodies, decoy receptors, as well as novel strategies to target TAM ligands are being developed. This paper will review the biology of TAM receptors and their ligands with a focus on cancer, as well as evidence-based data for the continued pursuit of TAM/Gas6 inhibitors in clinical practice.

## 1. Introduction

The TAM receptor tyrosine kinases (RTKs) comprised of Tyro3 (synonyms; BYK, Dtk, RSE, Rek, Sky, Tif, or Etk-2), Axl (synonyms; ARK, UFO, JTK11, or Tyro7) and Mertk (synonyms; MER, RP38, c-Eyk, c-Mer, Tyro12), have been under intense study over the last several years for their involvement in the resolution of inflammation, autoimmunity, and most recently for their role in cancer progression and tumor immunology [1,2,3,4]. TAM receptors (TAMs) are defined as family members based on a series of highly conserved amino acids in the catalytic intracellular tyrosine kinase domain, as well as structural commonalities in the extracellular domains consisting of two tandem immunoglobulin-like domains (Ig1 and Ig2) followed by two tandem Fibronectin type 3 (FN III)-like domains [5]. The main ligands of TAMs are Growth arrest specific factor 6 (Gas6) and Protein S (Pros1), similar proteins that require vitamin K (Vit-K) dependent γ-carboxylation for their ability to activate the tyrosine kinase activities of TAMs [6,7].

Unlike many RTKs that are embryonic or perinatal lethal when targeted by genetic ablation, TAMs are nonessential for embryogenesis and early development. As such, single, double or even triple Tyro3, Axl, and Mertk knockouts (KOs) are viable into puberty without catastrophic developmental or perinatal defects [8,9,10]. At around 4–5 weeks of age, which coincides with early post-puberty in murine development, TAM-deficient mice exhibit spontaneous splenomegaly and enlarged lymph nodes, a common manifestation of chronic and affirmative lymphoid activation [10]. At the cellular level, TAM deficiency results in spontaneous activation of both dendritic cells (DCs) and macrophages, which ultimately leads to skewed systemic cytokine imbalance in favor of inflammatory cytokines, circulating auto-antibodies (i.e., anti-double stranded DNA and anti-histone), and glomerulonephritis, the latter reminiscent of human Systemic Lupus Erythematosus (SLE) [11,12,13]. Although the autoimmune phenotype of triple TAM KO is most penetrant, the Mertk kinase-dead (kd) mice (essentially a KO) recapitulates some aspects of the triple KO in terms of an autoimmune phenotype indicating that not all functions of TAMs are compensatory [12]. For example, Mertk KO mice show notable defects in the clearance of apoptotic cells, resulting in the release of secondarily necrotic cell-derived material (SNECs) that persist and can induce anti-ANA antibodies and glomerulonephritis as noted above [12]. Such studies exploring phenotypes of knockout mice, as well as adoptive bone marrow transplantation studies in irradiated syngeneic mice (both transplanting TAM mutant hematopoietic cells into WT mice as well as WT hematopoietic cells TAM mutant mice), have led to a conceptual idea in the field that TAMs act as pleiotropic negative regulators of immune responses to maintain homeostasis and peripheral tolerance [13,14]. Consequently, as a result of uninhibited activation of innate immune cells (macrophages, natural killer (NK) cells, and DCs) without negative feedback inhibition, TAM ablation can lead to the generation and activation of self-reactive lymphocytes and hallmarks of autoimmunity [12]. Collectively, recent dogma holds that TAMs function, at least in part, by acting as “dampening” receptors at the interphase between innate and adaptive immunity therefore controlling the strength of immune signals to T effector functions.

Teleologically, this idea that TAMs act at the boundaries between innate and adaptive immunity is also noted by the fact that TAMs have evolved relatively late in evolution, and appear to have auxiliary fine-tuning roles, but not essential roles in controlling homeostasis in complex metazoans. Indeed, by sequence homology analysis, there are no homologs of TAMs in *Caenorhabditis elegans* or *Drosophila melanogaster* organisms that have relatively simple innate immune systems [3,15,16,17]. As noted below, the co-evolution of TAMs with more specialized and complex adaptive immune systems, possibly that have not yet been “hard-wired” by genetic redundancy, may make TAMs attractive targets in oncology and/or infectious diseases.

## 2. Expression of TAMs

While TAM receptors have arguably been best studied via their expression on myeloid-derived hematopoietic cells, such as DCs, macrophages, and NK cells, it is also clear that TAMs are broadly expressed in several cells and tissues, an observation that has been extensively discussed in several recent reviews [2,5,15,18]. However, it is also noteworthy that under dynamic inflammatory and hormonal conditions, the expression of TAMs in myeloid-derived DCs and macrophages, as well as non-myeloid cells such as epithelial cells, are tightly regulated at both the protein and mRNA level [3,18,19]. A good example of this type of dynamic and differential regulation of TAMs is offered by reciprocal regulation of Mertk and Axl under tolerogenic versus inflammatory conditions. In this capacity, tolerogenic signals (i.e., immunosuppressive glucocorticoids) induce transcription of Mertk [20,21], while simultaneously suppressing Axl transcription [22]. In contrast, inflammatory signals such as IFN-γ or poly (I:C) up-regulate Axl and simultaneously suppress Mertk expression [13,23]. In addition to transcriptional regulation by extracellular signals that impinge on transcription, TAMs can be post-transcriptionally regulated by micro-RNAs [24,25], as well as regulated at the level of protein by proteolytic processing (receptor shedding) [26,27] in addition to ligand-mediated ubiquitin-dependent protein degradation [28]. Clearly, much is still to be learned with respect to the complex regulation of TAMs under different physiological conditions. The development of TAM reporter mice, whereby transcriptional regulation of TAMs can be concomitantly monitored would be a welcome advance to query TAM regulation in vivo.

In cancer cells, overexpression of TAMs have been observed in a wide array of hematological and epithelial malignancies that include leukemia’s [29,30], non-small cell lung cancer (NSCLC) [31], glioblastoma [32], melanoma [33], prostate cancer [34,35], breast cancer [36,37], colon cancer [38,39], gastric cancer [40], and others. In some tumors, including acute myeloid leukemia (AML), NSCLC, and melanoma, overexpression of one or more of the TAMs (i.e., Mertk or Axl) and activation of tyrosine kinase activity can directly transform cells. Moreover, TAMs can also induce epithelial to mesenchymal transition (EMT), metastatic dispersal and chemo-resistance to targeted therapeutics [41,42]. Although much still needs to be learned with respect to mechanisms by which TAMs are up-regulated, this appears to be multi-factorial. These include observations that Axl and Tyro-3 promoters contain HIF1α-responsive elements that bind HIF1α and activate transcription under hypoxia and metabolic stress [43]. Moreover, the Mertk promoter contains steroid-responsive elements that activate transcription in estrogen and androgen-positive tumors [20]. Consistent with the above arguments on the centrality of TAMs in cancer, TAM ablation, by pharmacological or genetic means, decreases tumor growth and often resets chemo-sensitivity [3,44,45]. Clearly, the frequency at which TAMs are overexpressed in a wide range of human cancers has led to great ferment in the field to generate anti-TAM therapeutics.

In addition to expression in cancer cells, there is also growing appreciation that TAMs are also expressed on a variety of myeloid cells that contribute to the pathological milieu of the tumor microenvironment. Macrophages, DCs, myeloid-derived suppressor cells (MDSCs), NK cells, platelets, mast cells, and cancer-associated fibroblasts (CAFs) express TAMs, and appear to drive inhibitory signals that can lead to suppression of host anti-tumor immune responses. In support of this idea, recent studies have shown that Mertk ablation on tumor leukocytes in tumor bearing mice suppress both tumor growth and progression (metastasis) by a mechanism that depends, at least In part, by increasing in pro-inflammatory cytokines, polarizing M2 to M1 macrophages, and increasing cytotoxic T cells in the tumor microenvironment [46]. Moreover, additional studies showed that TAM expression on NK cells also exerts similar inhibitory signals in the cancer microenvironment via the E3 ubiquitin-ligase Cbl-b [47]. In these latter studies, these investigators identified TAMs as ubiquitylation substrates for Cbl-b and that pharmacological inhibitors of TAMs (or the TAM/Cbl degradation axis) could markedly inhibit tumor growth and metastasis. While additional studies are required to understand the dynamic expression of TAMs in totality within the tumor microenvironment, particularly with respect to immunogenic versus poorly immunogenic tumors, such data nonetheless support the general idea that TAM receptors will have an equally important role as checkpoint inhibitors on innate immune cells, possibly acting akin to negative regulators of the PDL1/PD1 axis.

While TAMs do not appear to be functionally expressed on lymphoid cells (T and B cells), recent studies have shown that activated T effector cells express Pros1 (and concomitantly expose phosphatidylserine (PS)) and can negatively feedback to regulate DC activation [48]. Such studies showed that genetic ablation of Pros1 on mouse T cells led to increased expression of co-stimulatory molecules on DCs, and enhanced immune responses to T cell-dependent antigens. Such data elegantly show that negative feedback can occur from T cells back to antigen presenting cells to limit immune responses, and support further research examining anti-Tyro3 and/or anti-Pros1 antagonists as direct checkpoint inhibitors in cancer therapeutics. Taken together, the aforementioned discussion suggest that TAMs may act as dual tumorigenic gene products, first by acting as direct drivers of tumor growth, and second by acting as inhibitory receptors in the tumor microenvironment that suppress host immunity.

## 3. TAM Ligands

The best-studied ligands for TAMs are the Vit-K modified γ-carboxylated proteins Gas6 [49,50,51] and Pros1 [52]. Gas6 and Pros1 share ~44% amino acid sequence homology, and have analogous domain organizations consisting of an N-terminal γ-carboxyl-glutamic acid (Gla) domain, 4 tandem Epidermal Growth Factor (EGF)-like repeats, and a C-terminal Sex Hormone-Binding Globulin-like region (SHBG), the latter consists of 2 Laminin G (LG) repeats [49,53].

Both Gas6 and Pros1 are exquisitely dependent on Vit-K mediated post-translational modifications for activity as TAM ligands which promotes γ-carboxylation of multiple glutamic acid residues in the N-terminal Gla domain (There are 11 highly conserved glutamic acid residues in the Gla domains of each ligand) [54]. Indeed, in vitro biochemical binding studies, as well as cell-based receptor activation studies, have shown that non-γ-carboxylated Gas6 (expressed in Warfarin-treated producer cells) [54,55] or Gas6 without Gla domain (Gla-less Gas6) [22] retain their ability to bind TAMs (with similar affinities), but prevent TAM activation. Although an exact biophysical or structural basis for why non-γ-carboxylated Gas6 binds but does not activate TAMs is not yet available, one possibility is that N-terminal γ-carboxylation allows for ligand-induced conformational changes and/or dimerization as a pre-requisite for receptor activation. Furthermore, it is well known that γ-carboxylation facilitates Gla domain binding to calcium that in turn allows its interaction with the anionic phospholipids, such as PS; which is known to be externalized on apoptotic cells, apoptotic blebs, exosomes, stressed tumor cells and vasculature, and on enveloped virus [56,57,58,59]. However, as noted above, the exact mechanisms by which PS/Gla interactions at the N-termini are communicated to the LG domain for ligand binding and conformational-induced receptor dimerization awaits further experimentation.

**Figure 1:**
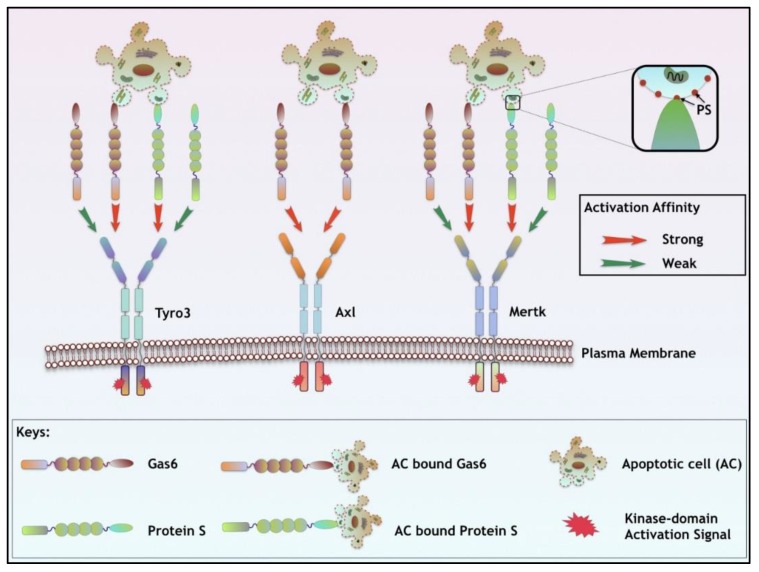
TAM receptor activation by Gas6, Pros1, and PS-positive apoptotic cells. TAM receptors exhibit differential activation by their ligands, Gas6 and Pros1. While Gas6 preferentially activates Axl and to lesser extent Tyro3 and Mertk; Pros1 does not activate Axl and is specific for Mertk and Tyro3. However, in the presence of externalized phosphatidylserine (PS) on the surface of apoptotic cells, stressed tumor vasculature, or PS- positive tumor exosomes, Gas6 and Pros1 mediated activation of TAMs is enhanced. After ligand binding, TAMs undergo subsequent dimerization and auto-phosphorylation of catalytic tyrosine kinase domain leading to downstream effector pathways.

While both Gas6 and Pros1 share common features of domain organization and both require γ-carboxylation for their activity as TAM ligands, they have differential specificities and affinities to Tyro3, Axl, and Mertk (Figure 1). In this capacity, while Gas6 has a high affinity for Axl (nM) and significantly lower affinity Tyro3 and Mertk (uM), Pros1 has a preference to Tyro3 and Mertk but does not activate Axl (Table 1) [18,54,60]. However, in the presence of externalized PS, both Gas6 and Pros1 hyper-activate Mertk and Tyro3 to intensify TAM signaling. Finally, it is noteworthy that in human plasma, Pros1 is detected at significantly higher concentrations (0.30 μM/L) [53] (approximately 1000 times higher), compared to Gas6 (0.16 to 0.28 nM/L) [61]. This difference may be explained, at least in part, by observations that Pros 1 also has an important role in the anti-coagulation pathways where it functions as a co-factor for Protein C during the inactivation of Factors Va and VIIIa [53,62]. Indeed, inherited Pros1 deficiency (in the Pros1 gene) leads to enhanced deep vein thrombosis and risk for embolism [63]. In contrast, loss of the Gas6 gene (by gene ablation) in mice prevents both venous and arteriole thrombosis by inhibiting platelet activation [64].

## 4. Targeting Ligand/TAMs for Anti-Tumor Therapeutic Response

Due to the centrality of TAM expression, both intrinsically on tumor cells as well as on resident and infiltrating immune cells that comprise the tumor microenvironment, there is a great effort to generate both selective TAM inhibitors, as well as pan-TAM inhibitors expected to target synergistic TAM functions in the entire tumor milieu. To date, at least four main classes of inhibitors are under consideration that include: (i) classical small molecule tyrosine kinase inhibitors (TKIs), (ii) soluble ectodomain receptors (so-called “decoy receptors”), (iii) antagonistic therapeutic antibodies, and (iv) direct and indirect Gas6 inhibitors.

In the case for TKIs, a number of TAM specific small molecule inhibitors are currently in pre-clinical and clinical development, and this has been discussed in several recent reviews. Among the earliest TAM-specific small molecule inhibitors is BGB324 (R428), a selective Axl inhibitor [37,65] currently in clinical development and of which Phase 1 trials were successfully completed in patients with acute myeloid leukemia (AML) and myelodysplastic syndrome (MDS). Similarly, a number of selective Mertk inhibitors are also under preclinical development including UNC569 (acute lymphoblastic leukemia (ALL)) [66], UNC1062 (metastatic melanoma) [33], UNC1666 (AML) [67] and UNC2025 (ALL and AML) [68]. In addition, several pan-TAM inhibitors are under development including 6g (preclinical) [45], BMS-777607 (phase I/II) [69,70], and LDC1267 (preclinical) [47]. These latter inhibitors are designed to target a broader array of tyrosine kinases (including pan-TAMs and other kinases such as c-Met) and may have fortuitous clinical utility. As a hypothetical example, pan-TAMs TKIs might be expected to target Axl on tumor cells, Mertk on infiltrating M2 macrophages, and Tyro3 on immature DCs, to collectively inhibit cancer growth, polarize macrophages and maximize antigen presentation. The success and utility of pan-TAM inhibitors, in combination with other checkpoint inhibitors will likely depend on the tumor type, the pattern of TAM expression, and will require trial and error evaluation. Future studies examining TAM inhibition, in combination with PS targeting agents or anti-PD1/PDL1 checkpoint inhibitors, are expected to reveal novel and synergistic combinatorial therapeutics.

The second type of TAM antagonist that is under investigation are TAM “decoy receptors” or “ligand traps”, molecules expected to block TAM receptor activation by sequestering the ligands. The “super-binding” Axl decoy receptors have been engineered and studied by Cochran and colleagues to block the Gas6 induced Axl receptor activation by sequestering Gas6 [71]. Interesting, this approach may phenocopy the physiological process of membrane shedding, a regulated proteolytic cleavage of the ecto-domains of Axl or Mertk receptors by enzyme ADAM17 (metalloproteinase A dis-integrin and metalloproteinase protein 17) [72,73]. Indeed, in other settings, Mertk or Axl ecto-domain shedding can have a protective physiological outcome, for example in conditions of thromboembolism, whereby soluble Mertk sequesters Gas6 to inhibit platelet aggregation [27]. The shedding of the extracellular domain of Mertk receptor by macrophages and retinal pigment epithelial cells has been shown to function as a decoy and contribute to atherosclerosis and retinal phagocytosis [74,75]. Functionally, the role of soluble TAMs as a ligand trap for Gas6/Pros1 might be expected to increase inflammatory cytokine production that in turn could activate innate and adaptive immune response against tumors. Finally, a variety of anti-TAM therapeutic antibodies are under clinical development, defined as a broadly agonist or broadly antagonistic. While antagonistic antibodies might be expected to have therapeutic activity in cancer, agonistic antibodies might be important to mimic TAM activation and drive tolerance, for example, in SLE or other chronic inflammatory conditions [76].

## 5. Targeting Gas6 and General Ligand Inhibitors

Another promising area of research that appears to be gaining traction has focused on targeting TAM ligands, most prominently recent studies that aim to target Gas6. These efforts are based on recent observations that in the tumor microenvironment, both over-expression of TAMs and its ligands (Gas6 or Pros1) occur concomitantly. This axis can be both autocrine in nature (both TAMs/Gas6 co-expressed in tumor cells or in myeloid cells) or paracrine in nature (TAMs in tumor cells/Gas6 in myeloid cells) to augment TAM signaling in the tumor microenvironment. In support of a tumorigenic role of Gas6 in ectopic and orthotopic syngeneic mouse tumor models, recent studies showed that both growth of tumors and subsequent metastatic dispersal was abrogated in a Gas6 deficient mice (Gas6−/−) compared to wild-type mice. These findings suggested that in the tumor microenvironment, tumor cells can “educate” the infiltrating leucocytes (mainly tumor-associated macrophages) to produce elevated Gas6 as an amplification loop, which in turn promotes tumor growth [77]. This observation is further complicated by the fact that Gas6 is produced in a wide variety of effector cells, many of which contribute to the tumor microenvironment (Table 2). Recent studies employing Gas6 neutralizing antibodies, GMAB1 and GMAB2, shown to decrease tumor growth in the pancreatic ductal carcinoma by blocking autocrine Gas6-Axl signaling clearly support the rationale to target Gas6 [78].

Further evidence that targeting Gas6 may have therapeutic implications have emerged from recent studies showing an anti-tumor and anti-metastatic role of low—dose warfarin administration. While warfarin has been used for decades to block γ-carboxylation of proteins involved in coagulation to reduce the risk of thrombosis, the warfarin-induced inhibition of Gas6/Pros1 γ-carboxylation and blockage of TAM activation alluded to above may also provide an interesting and opportunist approach to target a wide range of Gas6/TAM dependent tumors. Indeed, low-dose warfarin treatment in mice decreases tumor growth and metastasis by blocking the Gas6 induced Axl receptor activation [55]. Whether injection of non-γ-carboxylated Gas6 (or Pros1) acts as a dominant negative traps or competitive inhibitors to prevent ligand-inducible activation of TAMs awaits further experimentation.

**Figure 2:**
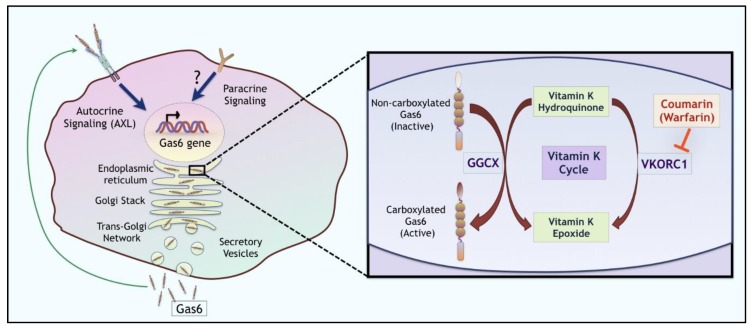
Post-translational modifications of Gas6 by γ-glutamyl carboxylation. Gas6 can be transcriptionally upregulated by autocrine or paracrine mechanisms. Following translation, un-carboxylated Gas6 (inactive) is activated by a series of enzymatic steps involving γ-glutamyl carboxylation via the vitamin-K-dependent enzyme γ-glutamyl carboxylase (Ggcx). The carboxylated (active) Gas6 from ER is transported to the Golgi apparatus through trans-Golgi network and then into secretory vesicles. The Vit-K epoxide reductase enzyme complex 1 (Vkorc1) then completes the Vit-K cycle by recycling this epoxide back to hydroquinone, which in turn serves as a co-factor in the Ggcx induced γ-carboxylation of Gas6 and Pros1. Warfarin, which functions as a direct inhibitor Vkorc1 prevents γ-carboxylation of Gas6 and Pros1 and prevents TAM receptor activation in the tumor microenvironment.

Concerning the above mentioned important role of Vit-K in Gas6/Pros1-mediated TAM activation, another component of the Vit-K/Gas6/Pros1 circuit warrants mention. This stems from the fact that Vit-K-dependent γ-carboxylation of Gas6/Pros1 is widely, if not ubiquitously, expressed in non-hepatic cells that include many of the cells that express Gas6 in the tumor microenvironment. Indeed, Vit-K-dependent γ-carboxylation of Gas6 and Pros1 along with many other Gla-containing proteins, most notably blood coagulation factors II, VII, IX, X, and protein C, is catalyzed by the enzyme γ-glutamyl carboxylase (Ggcx) that adds the γ-glutamyl moiety on the glutamic acid residues [98]. During this process, Ggcx oxidizes Vit-K hydroquinone and converts it to Vit-K 2,3 epoxide, while at the same time modifying glutamic acid (Glu) residues to γ-carboxy-glutamic acid (Gla) residues. This epoxide is then recycled back into hydroquinone via the enzyme Vit-K epoxide reductase complex-1 (Vkorc1), thus completing the Vit-K cycle and facilitating subsequent carboxylation events (Figure 2). The relevance of this pathway to cancer is of potential interest given overexpression of both Ggcx and Vkorc1 have been observed in several cancers, including both liver cancer and several adenocarcinoma’s that co-express Gas6 (Oncomine database). The co-overexpression of Gas6 and Vit-K modifying enzymes might be expected to produce hyper-activated TAM ligands (i.e., higher stoichiometry and/or density of glutamic acid modifications on Gas6/Pros1). These ideas raise several interesting queries that include (i) are extra-hepatic Ggcx and/or Vkorc1 targeted by low dose warfarin (known to have an anti-metastatic effect), (ii) do these enzymes, particularly when co-expressed with Gas6/Pros1, act as surrogate oncogenes for TAM receptors. Finally, these arguments also call in question the clinical use of Vit-K supplements for the treatment of osteoporosis and/or arterial calcification that could unmask a pro-oncogenic role for Gas6 and tumorigenesis, particularly in patients with latent tumors.

## 6. Synergistic Role of TAMs and of Dys-Regulated PS in the Tumor Microenvironment

**Figure 3:**
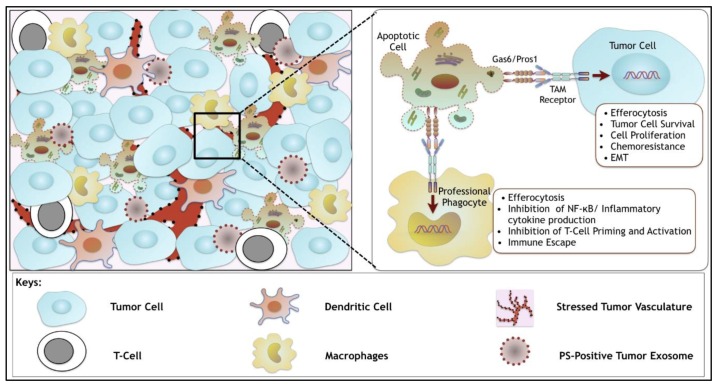
The role of TAM receptors in the tumor microenvironment. Various types of cells including tumor cells, macrophages, DCs, T-cells and apoptotic cells in addition to stressed tumor vasculature and phosphatidylserine (PS) positive tumor exosomes contribute to the tumor vasculature. In the tumor microenvironment, Gas6 binds to PS on the apoptotic cells and tumor exosomes and activates TAM receptors (TAMs) on tumor cells as well as on phagocytes such as macrophages, and DCs (professional phagocytes). Activation of TAMs on the tumor cells drive tumor growth and metastasis via downstream effector signaling leading to tumor cell survival, proliferation, chemoresistance and EMT phenotypes. On the other hand, activation of TAMs on the professional phagocytes leads to engulfment of apoptotic cells (efferocytosis), which in turn drives immune evasion by inhibiting T-cell priming and activation as well as via inhibition of NF-κB and inflammatory cytokine production. Hence, TAMs may act as dual tumorigenic gene products, first by acting as direct drivers of tumor growth, and second by acting as inhibitory immune receptors in the tumor microenvironment.

While the discussion above has focused mainly on the up-regulation of TAMs and TAM ligands (Gas6/Pros1) in the tumor microenvironment, a final important factor, namely the concomitant dysregulation of PS in the tumor microenvironment also warrants mention. Indeed, due to the hypoxia and other metexpressabolic stress, the high apoptotic indexes of apoptotic cells, and the release of tumor derived exosomes, up-regulation of the PS in the tumor microenvironment has been observed in several cancers [99,100]. As noted above, the interaction of PS with the overexpressed TAMs via its ligands, Gas6 and Pros1, on the tumor cells induce several tumorigenic phenotypes including tumor cell survival, proliferation, chemoresistance as well as tumor metastasis. On the other hand, PS interaction with TAMs on infiltrating myeloid derived phagocytes can promote PS-dependent efferocytosis, clearance of apoptotic cells [101], and elicit the production of immunosuppressive cytokines such as IL-10 and TGF-β (Figure 3). Indeed, recent studies provide an elegant example of this paradigm, whereby the massive apoptosis associated with post-partum mammary involution was associated with PS/Mertk-mediated efferocytosis and the production of “wound-healing” cytokines, including IL-4, IL-10, and TGF-β that induced epithelial to mesenchymal transition and metastasis of resident tumor cells [102]. Such observations may suggest that targeting TAMs together with PS (by PS targeting antibodies such as bavituximab) may have therapeutic benefit to limit PS mediated amplification of the Gas6/Pros1-TAM activation axis [103].

## 7. Conclusions

The recent characterization of TAM receptors as dual function oncogenic receptors on tumor cells and inhibitory receptors on immune cells involved in immune evasion has opened up an exciting new area of cancer biology where many questions remain unanswered (Box 1).

Box 1. **Unanswered questions in the TAM receptor field.**

- Are TAM receptors and their ligands (Gas6 and Pros1) up-regulated in poorly immunogenic tumors?
- Can TAM receptors and TAM ligand antagonist be used in combination with other check point inhibitors such as anti-PD1 and anti-CTLA-4?
- Can warfarin or non-γ-carboxylated Gas6 proteins be considered as adjuvant cancer therapeutics?
- Does over-expression of *Ggcx* and *Vkorc1* contribute to tumorigenicity of the tumor microenvironment?
- What is the role of non-γ-carboxylated TAM ligands (Tubby, TULP-1, Galectin-3 [104,105,106]) in the tumor microenvironment?
- What is the molecular mechanism(s) by which TAM post-receptor signaling drive anti-inflammatory and immune inhibitory signals?

Further studies aimed to better understand the dynamic regulation and expression TAMs in the tumor microenvironment will add new insights into how to systematically target TAMs, as well as to identify optimal combinations of TAM inhibitors with targeted therapies and checkpoint inhibitors. We await further pre-clinical and clinical data about how (TAMs/Ligands/PS) can be maximally exploited in cancer.

## Figures and Tables

**Table 1:** Properties of TAM ligands.

Ligand Properties	Gas6	Protein S
TAM Specificity	Axl >> Tyro3 > Mertk	Tyro3 > Mertk; Axl (ND)
TAM Specificity (In the presence of PS)	Tyro3 = Mertk >> Axl	Tyro3 = Mertk; Axl (ND)
Vit. K dependency	√	√
Plasma Concentration	0.16 to 0.28 nM/L	0.30 μM/L (~1000 times)

**Table 2:** Various Cell-types producing Gas6.

Cell Type	Reference
Adipose Tissue	[79]
Bone Marrow cells	[77,80,81,82]
Endothelial Cells	[6,79,80,82,83,84]
Epithelial Cells	[85]
Fibroblast	[6,79,86,87]
Hematopoietic cells	[10,19,81]
Hematopoietic stem cells	[88]
Hepatocytes	[89]
Macrophage	[77,90,91]
Mesangial cells (kidney)	[84]
Microglia Cells	[92,93]
Neuron	[94]
Pericytes	[83]
Plasma	[61,86,95,96]
Platelets	[64,80,82]
Stromal Cells	[85]
Vascular smooth muscle cell	[80,82,83,97]
